# The relationship between maternal-infant bonding and postpartum depression/anxiety: moderating effect of childhood psychological abuse and validation of the Mother-to-Infant Bonding scale (MIBS-8) in Arabic

**DOI:** 10.1186/s12888-024-05745-9

**Published:** 2024-04-17

**Authors:** Diane El Hadathy, Diana Malaeb, Souheil Hallit, Feten Fekih-Romdhane, Habib Barakat

**Affiliations:** 1 School of Medicine and Medical Sciences, Holy Spirit University of Kaslik, P.O. Box 446, Jounieh, Lebanon; 2 College of Pharmacy, Gulf Medical University, Ajman, United Arab Emirates; 3 Applied Science Research Center, Applied Science Private University, Amman, Jordan; 4 The Tunisian Center of Early Intervention in Psychosis, Department of Psychiatry “Ibn Omrane”, Razi Hospital, 2010 Manouba, Tunisia; 5 Faculty of Medicine of Tunis, Tunis El Manar University, Tunis, Tunisia; 6 Department of Obstetrics and Gynecology, Notre Dame des Secours University Hospital Center, Postal Code 3, Byblos, Lebanon

**Keywords:** Postpartum, Depression, Anxiety, Mother-infant bond, Child abuse

## Abstract

**Background:**

The emotional bond that a mother senses to her infant is essential to their social, emotional, and cognitive development. Understanding the level of mother-infant bonding plays an imperative role in the excellence of care. However, in Lebanon, there is a paucity of information about mother-infant bonding in the postpartum period. Given that Lebanese pregnant women constitute an important part of the population to look at, the objectives of the study were to (1) validate the Arabic version of the mother–infant bonding scale and (2) the relation between mother-infant bond and postpartum depression/anxiety; (3) the moderating effect of child abuse in the association between mother-infant bond and postpartum depression/anxiety.

**Methods:**

This cross-sectional study was conducted from September 2022 until June 2023, enrolling 438 women 4–6 weeks after delivery (mean age: 31.23 ± 5.24 years). To examine the factor structure of the mother-infant bond scale, we used an Exploratory-Confirmatory (EFA-CFA) strategy. To check if the model was adequate, several fit indices were calculated: the normed model chi-square (χ^2^/df), the Steiger-Lind root mean square error of approximation (RMSEA), the Tucker-Lewis Index (TLI) and the comparative fit index (CFI).

**Results:**

EFA was conducted on the first subsample. Three items were removed. The five items remaining loaded on one factor, which explained 73.03% of the common variance (ω = .91 / α = .90). After adding a correlation between residuals for items 2–7 and 5–8, fit indices of the CFA results were acceptable: χ2/df = 6.97/3 = 2.32, RMSEA = .068 (90% CI .001, .135), SRMR = .017, CFI = .996, TLI = .988. The interaction maternal-infant bonding by child psychological abuse was significantly associated with depression and anxiety respectively. At low, moderate and high levels of child psychological abuse, higher maternal-infant bonding scores (greater difficulty in bonding) were significantly associated with higher depression and higher anxiety respectively.

**Conclusion:**

This study provides, for the first time, a specific Arabic scale to assess mother-infant bonding reliably and validly. Furthermore, our study has suggested the existence of factors that have additive effects in potentiating the risk for depression and anxiety among Lebanese postpartum women, namely a history of psychological child abuse. Therefore, laborious awareness programs and healthcare services need to be implemented in order to prevent maternal mental health disorders from being unrecognized and left untreated.

## Background

Postpartum is a vulnerable period that presents a number of challenges to women. In fact, postpartum appears to be a potent trigger of mood disorders. The Diagnostic and Statistical Manual of Mental Disorders, 5th Edition (DSM-5), states that the “Postpartum Onset” (a class of major depressive disorders) is characterized by symptoms appearing 4–6 weeks after delivery. The most statistically and clinically relevant psychological complication related to giving birth to children is postpartum depression. About 10–15% of women giving birth may develop postpartum depression (PPD), with differences between population groups and geographical locations [1]. More specifically, postpartum depression symptoms include disturbances in appetite and sleep, energy loss, sensation of guilt, diminished attentiveness and suicidal thoughts. Recent findings showed a prevalence rate of 19.8% of postpartum depression after birth (between 19.5 and 20.0%) [2].

Perinatal anxiety is another vulnerability that women are prone to develop during pregnancy and after giving birth. Anxiety is experienced throughout pregnancy (antenatal) and/or the postpartum period [3]. Perinatal anxiety has been recognized as a reliable leading indicator of postpartum depression [4]. A study carried out by Miller et al. detected that depression can be the consequence of undiagnosed and untreated anxiety [5]. Another study found that women with an anxiety disorder during pregnancy are at three times greater risk of developing postpartum depression  [6]. In point of fact, depressive and anxiety disorders are strongly interrelated; for instance, Wisner et al. have detected the presence of comorbid anxiety disorders among almost two-thirds of women who screened positive for depression in the perinatal period [7]. Both perinatal depression [8–10] and perinatal anxiety [11, 12] can result in multiple detrimental consequences for mothers’ mental and physical health and well-being, as well as that of their infant. Therefore, research aiming at establishing correlates of perinatal depression/anxiety may represent a major and necessary step in developing effective, culturally applicable strategies and interventions. The present study proposes to focus on an important factor affecting postpartum depression/anxiety, which is maternal–infant bonding.

### The relationship between maternal-infant bonding and postpartum depression/anxiety

Maternal–infant bonding refers to a maternal-driven process of a passionate tie between a mother and her baby [13]. Bonding problems have three different manifestations: a) The mother may experience delays, ambivalence, or loss of emotional response and express dissatisfaction with her feelings toward her infant, b) A rejection of the infant may occur if the mother exhibits strong negative feelings about the child, such as regret, dislike, or hatred for his birth, and c) Pathological anger can manifest when the mother experiences anger that is controlled with difficulty or may have the drive to harm or kill the child or may lose control at a verbal level and shout and scream at the baby [14]. The development of the mother-infant bond is a major focus of obstetric, neonatal, and pediatric care. Many studies researched the connection between maternal bonding and postpartum depressive symptoms at 6 weeks after delivery or later. For example, in a study done in Japan, Nakano et al. (2019) found that postpartum depression measured 1 month after delivery is strongly associated with impaired maternal bonding measured 12 weeks after the delivery [15]. Furthermore, a systematic review conducted by McNamara et al. (2019) found that 15 of 19 research studies showed the evidence that postpartum depression is associated with maternal–infant bonding [16]. Moreover, an article published in 2020 demonstrated significant associations between bonding and maternal level of stress, anxiety and postnatal depression in Polish mothers [17]. However, the relationship between postpartum depression and mother-infant bonding is not well understood, however, multiple theories might explain this association; anger and rejection alter the ratio ACTH to cortisol levels, thus causing hypothalamic–pituitary–adrenal (HPA) axis dysfunction leading to increased vulnerability to mood disorders [18]. In addition, the lack of affection decreases monoamines, including serotonin leading to major depressive disorder. Relevant to postpartum depression, single nucleotide polymorphisms (SNPs) in tryptophan hydroxylase 2 gene (TPH2), an enzyme in the rate-limiting step in serotonin synthesis, has been associated with postpartum depression [19]. Human babies are born very dependent on their parents. They undergo huge brain development, growth and neuron pruning in the first two years of life. The brain development of infants (as well as their social, emotional and cognitive development) depends on a loving bond or attachment relationship with a primary caregiver, usually a parent and mainly the mother. A good initial bond is therefore more important than we could ever have imagined. Without such a factor, children are less likely to grow up to become happy, independent and resilient adults. Many pathologies have been reported in children who suffered neglect (an extreme form of insecure attachment) in their early years: reduced growth in the left hemisphere which may lead to associated increased depression risk for depression, Increased sensitivity in the limbic system which can lead to anxiety disorders and reduced growth in the hippocampus that could contribute to learning and memory impairments. However, in Lebanon there is lack of information on mother-infant bonding during the postpartum period because there is no Arabic validation tool to assess and diagnose this issue.

There are many variable manipulations and risk factors that can play a role on the association of mother-infant-bonding and postpartum depression/anxiety. According to study done in Japan [20], lower education level was associated with a higher prevalence of postpartum depression and related symptoms. A prospective Brazilian study [21] found that women with an unplanned pregnancy were 2.5 times more likely to have a depression during pregnancy and the postpartum period compared to women with a planned pregnancy. Women with low monthly income, less than a college education, unmarried, unemployed were 11 times more likely than women with no these socioeconomic risk factors to have clinically elevated depression scores at 3 months postpartum [22]. In addition, an observational study showed that the group that received epidural analgesia had lower pain scores, so a high correlation between epidural analgesia and lower depression levels was found [23]. The present study sought to contribute to the existing body of knowledge, by investing the role of childhood trauma in the pathways between maternal-infant bonding and postpartum anxiety/depression.

### Childhood trauma as a moderator on the association between maternal-infant bonding and postpartum distress

Child maltreatment refers to “all forms of physical and emotional ill-treatment, sexual abuse, neglect, and exploitation that results in actual or potential harm to the child’s health, development or dignity” [24]. Four main types of child abuse can be identified: physical, sexual, and psychological abuse (acts of commission), and neglect (act of omission in the care). Little is known about the relationship between maternal childhood maltreatment and postpartum mother-infant-bonding (MIB), controlling for the role of postpartum mental health. A cross-sectional study [25], published in August 2019, was the first to explore the association between diverse types of maternal childhood maltreatment and postpartum MIB, adjusting for postpartum mental health and it was found that almost 46% of the included women reported at least one type of childhood maltreatment with emotional neglect being most prevalent; 13% displayed at least mild postpartum depressive symptomatology and 20% scored above the 75th percentile for postpartum anxiety. In addition, that study showed that higher severity of maternal emotional neglect in childhood, higher levels of postpartum depression were significantly related to more postpartum MIB impairment.

Emotional abuse is particularly damaging to a child’s self-esteem and emotional wellbeing. Also known as psychological or verbal abuse, it is the most common form of child abuse. It can include constant rejection, hostility, teasing, bullying, yelling, criticism and exposure to family violence. Studies demonstrated that repeated incidents of psychological abuse build up over time resulted in mental health disorders (depression, anxiety, phobias) [26]. A study done on Norwegian women showed that women reporting abuse had an 80% increased risk of experiencing PPD as compared to non-abused women [27]. When the definition of abuse was narrowed to childhood abuse, the association was strongest in women who experienced childhood psychological abuse rather than sexual or physical abuse or neglect. Furthermore, adverse childhood experiences were associated with particular physiological changes and health risks that increase the susceptibility to anxiety [28, 29]. In addition, previous studies found an interaction between childhood trauma and oxytocin levels on bonding scores, suggesting a physiological response to early abuse that can have implications on mothers’ bonding perceptions. Prior maternal experiences, such as traumatic experience in childhood and adulthood, have been shown to negatively impact maternal-infant bonding [30]. Muzik et al. found that a sample of 97 African American and White mothers with childhood histories of neglect exhibited significant bonding impairment at 6 weeks, 4 months, and 6 months postpartum compared to healthy controls [31].

### Measures of maternal-infant bonding

Several observational assessment tools exist to evaluate attachment and interaction behaviors between mother and infant. For example, the Mother-to-Infant Bonding Scale (Taylor, 2005), the Maternal Postpartum Attachment Scale (Condon & Corkindale, 1998), and the Postpartum Bonding Questionnaire (PBQ) (Brockington, 2001). Despite the wealth of antenatal and postnatal measures, the psychometric properties of these tools remain poor and understudied. In order to have clinical and research utility, self-report measures must meet criteria for validity and reliability. The Mother–to-Infant Bonding Scale (MIBS-8) demonstrated sufficient evidence for structural validity, internal consistency and reliability with high quality of evidence. The MIBS-8 follows the current COSMIN guidelines, is short, quick, easy-to-administer. Moreover, the relative ease of administration means that these instruments can be more readily incorporated into large-scale studies and surveys, including those with multiple follow-ups. The MIBS-8 was originally developed based on the mother–infant bonding questionnaire (MIBQ) by Kumar (1997), consisting of nine items, namely feeling loving, resentful, neutral or nothing, possessive, joyful, dislike, protective, disappointed, and aggressive. Taylor et al. (2005) adapted the MIBQ by adding one item – feeling ‘scared or panicky’ – making the items 10 in number. Taylor’s MIBQ was adapted by Keiko Yoshida et al. (2012), who called it the MIBS-Japanese (MIBS-J). The confirmatory factors analysis conducted by Yoshida et al. (2012) produced 8 significant items, which were divided into 2 factors, namely the ‘lack of affection’ factors (items number 1, 4, 6, 8, and 10) and ‘anger and rejection’ factors (items number 3, 5, and 9) [32]. Higher scores reflect worse mother to infant bonding. The MIBS-8 has been validated in many languages, including Japanese [32], Chinese [33], English [33], Swedish [34], Tamil [35] and Indonesian [36]. However, no Arabic validation exists so far to the best of our knowledge.

For nearly three years, Lebanon has been assailed by the most devastating, multi-pronged crisis in its modern history. The unfolding economic and financial crisis that started in October 2019 has been further exacerbated by the dual economic impact of the COVID-19 outbreak, and the massive Port of Beirut explosion in August 2020. Of the three crises, the economic crisis has had by far the largest (and most persistent) negative impact. The Spring 2021 Lebanon Economic Monitor found that Lebanon’s economic and financial crisis ranks among the worst economic crises globally since the mid-nineteenth century [37]. Furthermore, recent findings have showed that domestic violence against women in the Lebanese population has increased [38] as well as child abuse, neglect and household dysfunction [39]. All of these resulted in mental health issues among Lebanese population [40, 41] including depression and anxiety. According to a recent study, depression and anxiety rates in Lebanese postpartum women seemed to be higher compared with other countries, which may in part be due to differences in regional, social and environmental culture [42].

The emotional bond that a mother senses to her infant is essential to their social, emotional, and cognitive development. Understanding the level of mother-infant bonding plays an imperative role in the excellence of care. However, in Lebanon, there is a paucity of information about mother-infant bonding in the postpartum period. Furthermore, few studies have examined the importance of childhood maltreatment in the etiology of postpartum depression/anxiety worldwide, and no previous studies have examined the moderating effects of childhood maltreatment on the mother-infant bonding to our knowledge.

The development of the relationship between the mother and the newborn is the most important process in the puerperium, and it is the topic of investigation for researchers since late 1960 [43]. The impact of difficulties in mother-infant bonding has been also well studied and documented in the literature [44]. It is crucial to early diagnose any disordered bonding as early as possible, considering that mothers are usually not aware about bonding problems they might have. For this reason, many instruments have been designed to screen for mother-infant bonding issues. It is also worth noting that none of the bonding instruments were validated to the Arabic language.

Given that Lebanese pregnant women constitute an important part of the population to look at, the objectives of the study were to (1) validate the Arabic version of the mother–infant bonding scale and (2) the relation between mother-infant bond and postpartum depression/anxiety; (3) the moderating effect of child abuse in the association between mother-infant bond and postpartum depression/anxiety. We hypothesized that the Arabic MIBS would show adequate validity and reliability, and we propose that psychological child abuse enhances the cross-sectional correlation between impaired mother-infant bonding and postpartum depression/anxiety.

## Methods

### Study design

This cross-sectional study has been conducted from September 2022- June 2023. An anonymous questionnaire has been disseminated to women who has recently delivered (4–6 weeks after delivery) in different hospitals in Lebanon. Snowball sampling and respondent-driven sampling techniques were implemented for data collection. A soft copy of the questionnaire was created using Google Forms and sent to participants via hospital’s emails, social media platforms and messaging applications. Prior to participation, study objectives and general instructions were thoroughly explained. Participants have been informed that this study is anonymous and participation is voluntary.

### Minimal sample size calculation

The G-power software calculated a minimal sample of 380 women, based on an F test and a 5% multiple regression R^2^ deviation from zero, α error = 5%, power = 80%, and a maximum of 16 factors to be included in the multivariable analysis.

### Questionnaire

The first question is asking the consent (if she is willing to participate in our study).

The first part of the questionnaire consisted of questions that were linked to factors associated with postpartum depression/anxiety according to the literature review: age, marital status, education level, occupation (employed or unemployed), if planned pregnancy, pregnancy mode, epidural injection, pain during pregnancy, insurance, parity, household crowding index reflecting the socioeconomic status and financial burden.

The second part consisted of the scales used in the study:

-We used the Edinburgh Postnatal Depression Scale (EPDS) to screen for the possible presence of postpartum depression. It is the standardized tool used postnatally to quantify the severity and establish an estimation of postpartum depression [45]. EPDS is a valid 10-question scale that is valuable for identifying the potential risk of depression following childbirth and an effective screening tool that demonstrates sensitivity and high reliability. Regarding the scoring of the EDPS questionnaire, the following is used: answers are scored on a scale from 0 (not at all) to 3 (as much as I ever did). Therefore, the total score ranges from 0 to 30, with a score of 11 or more deemed positive for postpartum depression. On the total EPDS score, the threshold value ≥ 11 is deemed a relevant diagnostic criterion in order to diagnose appropriately postnatal depression during the post-delivery time range of 4–6 weeks.

This questionnaire also contained the “Perinatal Anxiety Screening Scale” (PASS) to detect the severity of perinatal anxiety. The PASS is a trustworthy 31-item self-reported questionnaire for postpartum and antenatal women to screen for anxiety. It distinguishes between high and low anxiety disorder risk by measuring specific anxiety symptoms. The mother scores these symptoms by indicating their frequency over the last month. The scale is as follows: 0 (not at all), 1 (sometimes), 2 (often) and 3 (always). Scores between 0 and 20 indicate the absence of anxiety symptoms, scores between 21 and 41 indicate mild-to-moderate symptoms, and scores between 42 and 93 indicate severe symptoms. High and low anxiety disorder risks were separated with a cut-off score of 26. It is important to mention that these two scales (EPDS and PASS) are validated in Arabic language [42].

The Child Abuse Self-Report Scale (CASRS): The Arabic version of the 12-item Child Abuse Self Report Scale mirrors the original four factors captured by the original CASRS. The scale showed a good internal consistency as evidenced through McDonald’s ω values ranging from 0.87 to 0.93 for the four subscales; and configural, metric, and scalar invariance across gender [46]. Inter-item correlations and Item-total correlations were obtained from the study using the Arabic 38-item CASRS. It contained 12 items divided into four categories of child abuse: psychological (3 items), neglect (3 items), physical (3 items) and sexual (3 items). The responses are reported as follows: 0 = Never 3 = Always. Higher scores indicate more abuse in all subscales.

The Mother–infant bonding scale (MIBS-8): This scale was translated to Arabic in the purpose to be validated. It is composed of 8 items, divided into 2 factors, namely the ‘lack of affection’ factors (items number 1, 4, 6, 8, and 10) and ‘anger and rejection’ factors (items number 3, 5, and 9). Higher scores reflect worse mother to infant bonding. A protocol of initial translation followed by a back translation was adopted to translate all English scales used to Arabic. The initial translation from English to Arabic was carried out by a mental health professional, after which a different specialist translated this version in Arabic back again to English. To ensure the reliability of both translations at the end of the translation protocol, both English versions were compared. This comparison yielded no significant difference between the versions. The Arabic version was pilot tested on 30 pregnant women.

### Statistical analysis

There were no missing responses in the dataset. To examine the factor structure of the mother-infant bond scale, we used an EFA-to-CFA strategy [47]. To ensure adequate sample sizes for both EFA and CFA, we split the main sample using an SPSS computer-generated random technique; the two subsamples consisted of 150 and 288 participants respectively. We computed a principal-component EFA with the first subsample using the SPSS v.26 software. We verified all requirements related to item-communality (≥ 0.3) ( [48], anti-image correlations (≥ 0.50) [49], and item-total correlations (≥ 0.15) [50]. The Kaiser–Meyer–Olkin (KMO) measure of sampling adequacy (which should ideally be ≥ 0.80) and Bartlett’s test of sphericity (which should be significant) ensured the adequacy of our sample [51]. Item retention was based on the recommendation that items with “fair” loadings and above (i.e., ≥ 0.4).

We then used data from the second subsample to conduct a CFA using the SPSS AMOS v.29 software. Therefore, we assumed a minimum sample of 160 participants needed to have enough statistical power based on a ratio of 20 participants per one item of the scale, which was exceeded in our sample. Our intention was to test the original model of the mother-infant bond scale (i.e., a unidimensional model) and, if divergent, the model extracted from our EFA. The analysis was done using the maximum likelihood estimation method. To check if the model was adequate, several fit indices were calculated: the normed model chi-square (χ^2^/df), the Steiger-Lind root mean square error of approximation (RMSEA), the Tucker-Lewis Index (TLI) and the comparative fit index (CFI). Values ≤ 5 for χ^2^/df, and ≤ 0.08 for RMSEA, and 0.90 for CFI and TLI indicate good fit of the model to the data [52]. Multivariate normality was verified through the Bollen-Stine bootstrap *p* = 0.232. We considered adding a correlation between residuals of items in case the modification indices were high. Additionally, evidence of convergent validity was assessed in this subsample using the average variance extracted (AVE), with values of ≥ 0.50 considered adequate [53].

Composite reliability in both subsamples was assessed using McDonald’s ω and Cronbach’s α, with values greater than 0.70 reflecting adequate composite reliability. The depression and anxiety scores were considered normally distributed since their skewness and kurtosis values varied between -1 and + 1 [54]. The Student t was used to compare two means, ANOVA test between three means and the Pearson test to correlate two continuous variables. The moderation analysis was conducted using PROCESS MACRO (an SPSS add-on) v3.4 model 1 [55], taking mother-infant bond scores as the independent variable, childhood trauma dimensions as moderators and depression/anxiety as dependent variables. Results were adjusted over all factors that showed a *p* < 0.25 in the bivariate analysis. *P* < 0.05 was deemed statistically significant.

## Results

### Sociodemographic and other characteristics of the sample

Four hundred thirty-eight mothers participated in this study, with a mean age of 31.23 ± 5.24 years and 83.1% with a university education level. Other descriptive statistics of the sample can be found in Table 1.

**Table 1:** Sociodemographic and other characteristics of the sample (*n* = 438)

Variable	n (%)
Marital status	
Single	49 (11.2%)
Married	389 (88.8%)
Education level	
Secondary or less	74 (16.9%)
University	364 (83.1%)
Occupation	
Unemployed	189 (43.2%)
Employed	249 (56.8%)
Desired/intended pregnancy	
Yes	342 (78.1%)
No	96 (21.9%)
Pregnancy method	
Natural	388 (88.6%)
IVF	50 (11.4%)
Delivery method	
Normal	193 (44.1%)
C-section	245 (55.9%)
Epidural injection	
No	130 (29.7%)
Yes	308 (70.3%)
Pain during pregnancy	
No	222 (50.7%)
Yes	216 (49.3%)
Insurance	
No	88 (20.1%)
NSSF	183 (41.8%)
Private	167 (38.1%)
	**Mean ± SD**
Postpartum depression	13.61 ± 8.08
Postpartum anxiety	37.76 ± 27.11
Mother-to-infant bonding	2.72 ± 3.54
Age (years)	31.23 ± 5.24
Age at marriage (years)	26.63 ± 3.93
Number of children	1.73 ± .94
Household crowding index	1.12 ± .51
Perceived financial burden	8.04 ± 2.13
Childhood trauma—Psychological abuse	1.34 ± 2.46
Childhood trauma—Neglect	3.27 ± 2.76
Childhood trauma—Physical abuse	.81 ± 1.78
Childhood trauma—Sexual abuse	.84 ± 1.77

### Exploratory factor analysis

EFA was conducted on the first subsample. Prior to the analysis, we confirmed the suitability of the data via adequate KMO (= 0.873) and the *p* value of Bartlett’s test of sphericity (*p* < 0.001). We used the promax rotation since the anti-image values were above 0.8. Communalities were also above 0.3, indicating the adequacy of the analysis. Items 1 and 4 were removed at first because they loaded on both factors. We re-conducted the analysis on the remaining 6 items; the same conditions described in the first analysis were verified; item 6 was removed since it did not load on any factor. Five items remained; the third analysis showed that the Bartlett’s test of sphericity, χ^2^(10) = 513.48, *p* < 0.001, and KMO (0.854) indicated that the items had adequate common variance for factor analysis. The results of the EFA revealed one factor, which explained 73.03% of the common variance. Those 5 items loaded on one-factor and showed an excellent internal consistency (ω = 0.91 / α = 0.90).

### Confirmatory factor analysis

CFA was conducted on the second subsample and indicated that fit of the unidimensional model from the EFA was modest: χ^2^/df = 37.33/5 = 7.47, RMSEA = 0.150 (90% CI 0.107, 0.197), SRMR = 0.026, CFI = 0.970, TLI = 0.940. The modification indices between residuals of items 2–7 and 5–8; therefore, we added a correlation between those residuals. Consequently, fit indices improved and were acceptable: χ2/df = 6.97/3 = 2.32, RMSEA = 0.068 (90% CI 0.001, 0.135), SRMR = 0.017, CFI = 0.996, TLI = 0.988. The standardized estimates of factor loadings were all adequate (see Table 2). The convergent validity for this model was adequate, as AVE = 0.68. The internal consistency was also excellent (ω = 0.92 / α = 0.91).

**Table 2:** Factor Loadings Derived from the Exploratory Factor Analyses (EFA) in the First Subsample, and Standardized Estimates of Factor Loadings from the Confirmatory Factor Analysis (CFA) in the Second Subsample

Item	EFA	CFA
2. Resentful	.82	.75
3. Neutral or felt nothing	.74	.66
5. Dislike	.90	.86
7. Disappointed	.92	.91
8. Aggressive	.87	.91

### Bivariate analysis of factors associated with postpartum depression/anxiety

The results of the bivariate analysis of factors associated with postpartum depression/anxiety are summarized in Tables 3 and 4. The results showed that higher depression and anxiety were seen in mothers who were single, had a secondary level of education or less, were unemployed, did not want the pregnancy, got pregnant via IVF methods, did not receive epidural injection, had pain during pregnancy and did not have insurance coverage. Furthermore, higher depression and anxiety scores were correlated with higher mother-infant bond scores (worse bonding), higher household crowding index, financial burden, neglect, psychological, physical and sexual abuse. Finally, older age, older age at marriage and a higher number of children were significantly associated with lower levels of depression and anxiety.

**Table 3:** Bivariate analysis of factors associated with postpartum depression/anxiety

	**Depression**				**Anxiety**			
**Variable**	**Mean ± SD**	***t / F***	***df***	***p***	**Mean ± SD**	***t / F***	***df***	***p***
Marital status		14.72	436	** < .001**		16.46	436	** < .001**
Single	26.71 ± 4.22				85.00 ± 15.48			
Married	11.96 ± 6.85				31.81 ± 21.93			
Education level		8.39	436	** < .001**		8.97	436	** < .001**
Secondary or less	20.28 ± 10.31				61.46 ± 35.83			
University	12.25 ± 6.81				32.95 ± 22.12			
Occupation		5.03	436	** < 0.001**		4.33	436	** < .001**
Unemployed	15.78 ± 8.97				44.08 ± 31.00			
Employed	11.96 ± 6.91				32.97 ± 22.66			
Desired/intended pregnancy		7.30	436	** < .001**		7.78	436	** < .001**
Yes	12.20 ± 6.96				32.75 ± 22.90			
No	18.64 ± 9.69				55.60 ± 32.98			
Pregnancy method		3.88	436	** < .001**		4.95	436	** < .001**
Natural	13.08 ± 7.88				35.31 ± 25.86			
IVF	17.72 ± 8.52				56.78 ± 29.26			
Delivery method		.03	436	.975		1.81	436	.071
Normal	13.60 ± 7.69				35.16 ± 25.53			
C-section	13.62 ± 8.39				39.82 ± 28.18			
Epidural injection		6.75	436	** < .001**		7.12	436	** < .001**
No	17.43 ± 9.67				51.21 ± 32.83			
Yes	12.00 ± 6.70				32.09 ± 22.02			
Pain during pregnancy		8.16	436	** < .001**		7.59	436	** < .001**
No	10.70 ± 6.07				28.58 ± 18.65			
Yes	16.60 ± 8.79				47.20 ± 30.99			
Insurance		86.70	435	** < .001**		80.62	435	** < .001**
No	22.13 ± 7.78				65.58 ± 29.90			
NSSF	12.09 ± 6.82				32.85 ± 22.56			
Private	10.78 ± 6.35				28.49 ± 19.64			

Numbers in bold indicate significant *p* values

**Table 4:** Correlations matrix of continuous variables

	1	2	3	4	5	6	7	8	9	10	11	12
1. Postpartum depression	1											
2. Postpartum anxiety	.88***	1										
3. Mother-to-infant bonding	.76***	.78***	1									
4. Age	-.23***	-.22***	-.30***	1								
5. Age at marriage	-.12*	-.10*	-.17**	.65***	1							
6. Number of children	-.22***	-.24***	-.25***	.44***	-.06	1						
7. Household crowding index	.44***	.42***	.45***	-.16**	-.28***	.25***	1					
8. Perceived financial burden	.50***	.46***	.36***	-.03	.01	-.03	.36***	1				
9. Psychological abuse	.71***	.72***	.80***	-.39***	-.31***	-.22***	.54***	.35***	1			
10. Neglect	.71***	.67***	.69***	-.23***	-.23***	-.12**	.48***	.41***	.74***	1		
11. Physical abuse	.47***	.48***	.60***	-.33***	-.33***	-.16**	.45***	.21***	.68***	.52***	1	
12. Sexual abuse	.36***	.39***	.45***	-.22***	-.27***	-.11*	.34***	.14**	.52***	.40***	.82***	1

^*^p < .05; **p < .01; ***p < .001

### Moderation analysis

The details of the moderation analysis of the childhood trauma subscales taken as moderators in the association between mother-infant bond and depression/anxiety, are summarized in Table 5. All models were adjusted over all variables that showed a *p* < 0.25 in the bivariate analysis as follows: marital status, education level, occupation, desired pregnancy, pregnancy method, delivery mode, epidural injection, pain during delivery, insurance, age, age at marriage, number of children, perceived financial burden, and household crowding index. The interaction maternal-infant bonding by child psychological abuse was significantly associated with depression and anxiety respectively (Table 5). At low (Beta = 1.24; *p* < 0.001), moderate (Beta = 1.14; *p* < 0.001) and high (Beta = 0.93; *p* < 0.001) levels of child psychological abuse, higher maternal-infant bonding scores (greater difficulty in bonding) were significantly associated with higher depression. The same association was seen between maternal-infant bonding and higher anxiety at low (Beta = 4.35; *p* < 0.001), moderate (Beta = 3.92; *p* < 0.001) and high (Beta = 3.01; *p* < 0.001) levels of child psychological abuse (Table 6).

**Table 5:** Moderation analysis taking the mother-to-infant bonding as the independent variable, each child abuse subscale as a moderator and depression/anxiety as dependent variables

Moderator	Beta	*t*	*P*	95% CI
**Model 1: Depression as the dependent variable**				
**Model 1a: Psychological abuse as the moderator**				
Mother-infant bond	1.24	5.28	** < .001**	.77; 1.70
Psychological abuse	1.42	3.32	**.001**	.57; 2.27
Interaction mother-infant bond by psychological abuse	-.11	-2.08	**.040**	-.21; -.01*
**Model 1b: Neglect as the moderator**				
Mother-infant bond	1.10	2.91	**.004**	.35; 1.86
Neglect	.72	2.92	**.004**	.23; 1.21
Interaction mother-infant bond by neglect	-.01	-.12	.905	-.13; .12
**Model 1c: Physical abuse as the moderator**				
Mother-infant bond	1.35	6.15	** < .001**	.92; 1.79
Physical abuse	-.39	-.84	.405	-1.30; .53
Interaction mother-infant bond by physical abuse	-.02	-.39	.698	-.14; .10
**Model 1d: Sexual abuse as the moderator**				
Mother-infant bond	1.41	6.30	** < .001**	.96; 1.85
Sexual abuse	.19	.51	.609	-.55; .93
Interaction mother-infant bond by sexual abuse	-.08	-1.25	.213	-.20; .04
**Model 2: Anxiety as the dependent variable**				
**Model 2a: Psychological abuse as the moderator**				
Mother-infant bond	4.35	5.14	** < .001**	2.67; 6.03
Psychological abuse	6.09	3.95	** < .001**	3.03; 9.14
Interaction mother-infant bond by psychological abuse	-.47	-2.50	**.014**	-.84; -.10*
**Model 2b: Neglect as the moderator**				
Mother-infant bond	4.99	3.49	**.001**	2.15; 7.82
Neglect	1.33	1.42	.159	-.53; 3.19
Interaction mother-infant bond by neglect	-.17	-.72	.470	-.64; .30
**Model 2c: Physical abuse as the moderator**				
Mother-infant bond	4.56	5.59	** < .001**	2.94; 6.17
Physical abuse	-.51	-.30	.766	-3.90; 2.88
Interaction mother-infant bond by physical abuse	-.05	-.23	.820	-.50; .40
**Model 2d: Sexual abuse as the moderator**				
Mother-infant bond	4.87	5.93	** < .001**	3.24; 6.50
Sexual abuse	.29	.21	.833	-2.40; 2.97
Interaction mother-infant bond by sexual abuse	-.23	-1.04	.302	-.67; .21

^*^indicates significant moderation; numbers in bold indicate significant *p* values. Results adjusted over age, age at marriage, marital status, education level, occupation, wanted pregnancy, pregnancy method, delivery method, epidural injection, pain during delivery, insurance, number of children, financial burden and household crowding index

**Table 6:** Conditional effects of the focal predictor (mother-infant bond) at values of the moderator (child psychological abuse)

Child psychological abuse	Beta	*t*	*P*	95% CI
**Model 1: Depression as the dependent variable**				
Low (= .001)	1.24	5.28	** < .001**	.77; 1.70
Moderate (= .905)	1.14	5.30	** < .001**	.71; 1.56
High (= 2.841)	.93	4.51	** < .001**	.52; 1.34
**Model 2: Anxiety as the dependent variable**				
Low (= .001)	4.35	5.14	** < .001**	2.67; 6.03
Moderate (= .905)	3.92	5.06	** < .001**	2.39; 5.46
High (= 2.841)	3.01	4.05	** < .001**	1.54; 4.49

Numbers in bold indicate significant *p* values

## Discussion

The current study revealed that childhood psychological abuse had a moderating effect in amplifying the association between mother-to-infant bonding and postpartum depression/anxiety. In this study, we were also able to validate MIBS-8 in Arabic language. The preliminary psychometric properties of mother-infant bonding scale were satisfactory, showing that this scale was adequate to screen for mother-infant bond in Lebanese postpartum women.

### Validation of the Arabic MIBS-8

Our results supported the appropriate factorial validity and reliability of the MIBS-8. In terms of factorial validity, our results showed that scores are unidimensional, after removing three items (i.e., “loving”, “neutral or felt nothing”, “joyful”). These findings are comparable to the Japanese version, where authors failed to retain all eight items [56]. The three items removed reflected maternal lack of positive affection and intimacy towards the baby. We named these factors Lack of Affection. Indeed, fit of the unidimensional model of mother-infant bonding scale in the present study was adequate when tested using both EFA and CFA, with the latter achieving adequate fit with the need to omit the positively asked questions (1, 4, 6) and keep the 5 items concerning negatively asked questions. Cultural differences may represent a possible explanation for the present findings. Lebanese pregnant women are more prone to develop negative behaviors than pregnant women in other countries. According to the Lebanon Economic Monitor, the economic crisis in Lebanon is one of the worst in the world since the middle of 1800s. The simultaneous economic effects of the COVID-19 pandemic and the catastrophic explosion at the Port of Beirut in August 2020 have intensified the already-developing economic and financial crisis that began in October 2019. Raising a child under such stressful circumstances can hold may peculiarities and various implications on the nature of maternal-infant bond [57]. However, given the very limited number of studies conducted in Arab countries on the topic, more observational, cross-cultural psychometric research is needed to further examine whether bonding holds cross-culturally.

In addition, variables with high correlation are grouped together to reduce a large number of variables to a smaller set of composite factors to make them more understandable and easy to work with in our research. Item 1 (loving) and item 4 (joyful) load together because they are correlated with each other in representing good mother-infant bonding: love is a complex neurobiological phenomenon, involving oxytocin, vasopressin, dopamine, and serotonergic signaling [58]. After all, love is a joyful and useful activity that encompasses wellness and feelings of well-being. The psychological sense of love can be interpreted as referring to the satisfaction of a yearning, which may be associated with the obtaining of certain sensory stimulation so bringing back joy. Love therefore possesses a close connection with reward and pleasure phenomena like joy and happiness [59]. After adding a correlation between residuals for items 2–7 and 5–8, fit indices of the CFA results were acceptable: χ2/df = 6.97/3 = 2.32, RMSEA = 0.068 (90% CI 0.001, 0.135), SRMR = 0.017, CFI = 0.996, TLI = 0.988. Items 2 (resentful)-7 (disappointed) and items 5 (dislike)- 8 (aggressive) linked together. There is a link between dysfunctional cortico-limbic network and aggression [60]. People with higher levels of aggressive behavior exhibited more intense and frequent expressions of anger and resentment [61]. In fact, aggressive behaviors are a physiological response to disappointment and dissatisfaction [62]. The Arabic version showed a Cronbach’s alpha of 0.91. Evidence for acceptable internal consistency of the MIBS-8 has also been demonstrated in the original validation study (α = 0.7) and other previous linguistic validations, including the Japanese (α = 0.78) [63], Chinese (α = 0.73) [64] and Swedish (α = 0.78) versions. Accordingly, the preliminary results suggested this scale is adequate for the assessment of mother-infant bonding among Lebanese women.

### The association between maternal-infant bonding and postpartum depression/anxiety

Our results showed that greater difficulty in bonding was associated with more postpartum depression/anxiety. This is in agreement with previous findings that also found an association between postpartum depression/anxiety and poor bonding [65]. A study done in 2020 among Polish sample found significant associations between bonding and maternal level of stress, anxiety and postnatal depression [66]. In addition, studies showed that postpartum anxiety was significantly connected with an impaired bond [67].

### Childhood psychological abuse as a moderator

Our study found that child psychological abuse was significantly associated with impaired mother-infant bonding on one hand and postpartum depression/anxiety on the other hand. In accordance with previous studies, psychological or emotional abuse of a child can have more long-lasting negative psychiatric effects that either childhood physical abuse or childhood sexual abuse [68]. Adverse childhood experiences are likely an important determinant of maternal mental health difficulties, particularly postpartum depression/anxiety [69]. Women who experienced multiple adverse life events, including childhood psychological abuse, were found to be at an increased risk of postpartum depression, and were three times more likely to have postpartum depression compared to those that did not experience any adverse life events [70]. In this study, we also found a strong correlation between childhood abuse and maternal–infant bonding. This result is confirmed by other studies, suggesting that stress may have a negative effect on the maternal–infant bonding process [71]. In fact, many of the risk factors for postpartum depression fall under the umbrella of stress. In agreement with the clinical evidence for stress as a risk factor for postpartum depression, many of the animal models used to study postpartum depression utilize exogenous corticosterone or stress-based models. Exogenous corticosterone during pregnancy or lactation is sufficient to increase depression- and anxiety-like behaviors and induce deficits in maternal care in rodent models [72]. The hippocampus is altered in response to chronic stress, exposure to high glucocorticoids and in major depression in humans. High corticosterone reduced dendritic complexity and spines in the CA3 region of the hippocampus [73]. In addition, psychological abuse can significantly affect the whole trajectory of a person's life, to feel worthless, hopeless, helpless, and the loss of pleasure in daily activities as well as indulgence in self-suffering—all common symptoms of depression [74]. According to the World Health Organization (WHO), such stress can impair how a child’s nervous system and immune system develop. Because of this, survivors of childhood abuse are at an increased risk of physical and mental health problems. Children who experience emotional abuse are more likely to develop anxiety, depression, and memory issues. Psychological abuse can leave children with an unhealthy impression of family life, which they may carry into their own families later on. So, psychological abuse causes negative impacts on a society-wide level.

According to our study, psychological childhood abuse ( at any level: low, moderate or high levels) was associated with higher mother-infant bond scores (worse bonding) and more postpartum depression/anxiety. Consequently, there is a need to focus on child psychological abuse in order to have good mother-infant bond and prevent postpartum depression/anxiety. A better managing of child abuse leads to a better mother-infant bond which entails better postpartum mental health.

### Clinical implications

There is abundant room for further progress in determining other risk factors that may affect maternal bonding with a child such as social support and parental relationship quality. In general, understanding how maternal mental state is related to a mother’s perceptions of bonding and her behavior towards a child may help to provide various interventions for mothers in order to prevent potential negative outcomes. Therefore, laborious awareness programs and healthcare services need to be implemented in order to prevent maternal mental health disorders from being unrecognized and left untreated. Teaching mothers how to act with his/her infant to enhance mother-infant bonding, aiding mothers in the most effective ways of how to cope with the situations and feelings experienced in this period, resolving marital and family conflicts before conception, helping the mothers to draw a support plan, and having realistic expectations of birth and parenting, might be some of the ways to prevent postpartum disorders [75]. Furthermore, raising child abuse awareness by using graphics, social media posts, and sample proclamations can help attenuate the effects of bonding on postpartum depression/anxiety [76]. Finally, further studies need to confirm the psychometric properties of the Arabic MIBS such as measurement invariance and test–retest reliability. Future studies on bonding still need to adopt this scale in other arab countries in order to assess predictive validity of the Arabic MIBS.

### Limitations

There are some limitations to this study. Study variables were assessed in this study using self-reported instruments, and may therefore have been overestimated (e.g., [77, 78]). Future studies need to complete assessments using structured or semi-structured clinical interviews. Furthermore, a selection bias might be present due to the snowball technique, therefore, the sample is not representative of the whole population. In addition, different confounding variables not considered in this study (such as family or personal history of mental health disorder, insomnia, previous pregnancy loss, etc.) might play key roles in the relationship between our variables and their mediating effects should be tested in future studies.

## Conclusion

This study provides, for the first time, a specific Arabic scale to assess mother-infant bonding reliably and validly. We hope that the Arabic MIBS will facilitate routine screening for signs and symptoms of impaired mother-infant bonding among women who are predisposed to developing postpartum depression/anxiety. This screening is a necessary step towards appropriate and personalized prevention and treatment interventions. Since this represents the first study to explore the psychometric characteristics of the Arabic version of MIBS, findings should be considered tentative pending the outcome of future investigations in other arab countries. Furthermore, our study has suggested the existence of factors that have additive effects in potentiating the risk for depression and anxiety among Lebanese postpartum women, namely a history of psychological child abuse. Our study shows the moderating effects of childhood maltreatment on the association between mother-infant bonding and postpartum depression/anxiety. Understanding the risk factors related to postpartum depression/anxiety may help predict other factors to be tested in future studies for the purpose to increase the wellbeing and protect both the mother and the infant by implementing screening programs targeting women at risk for postpartum depression/anxiety.

## Data Availability

All data generated or analyzed during this study are not publicly available due the restrictions from the ethics committee. Reasonable requests can be addressed to the corresponding author (SH).
